# The Regius Professorship of Medicine at Oxford

**Published:** 1904-01-16

**Authors:** 


### The Regius Professorship of Medicine at Oxford.

The resignation of Sir John Burdon Sanderson teaches us how little changed is the mind of man from one generation to another. Jocelin of Brakelond has left to us an account of the talk which preceded the election of Samson, in 1182, to be abbot of the monastery of St. Edmund at Bury St. Edmunds. His account is one of the few glimpses we get of mediæval cloister life, and no translation renders the vivacity of the *dixit quidam de quodam* with which he prefaces the chatter going on about the election. So is it at the present time with the Oxford graduates of medicine scattered throughout England. Like the monks they are whole-heartedly desirous of obtaining the very best man for the post, and as then the Bishop of Winchester was the intermediary between them and Dominus Rex, so now Mr. Balfour occupies that important position. At a recent meeting of the Oxford graduates who are teachers in the medical schools at London, a document was drawn up and signed putting forth the qualities required to make an ideal Regius Professor—"A physician of great experience in clinical work and clinical teaching; thoroughly familiar with the system of instruction carried out at the large medical schools and with all the details of the students' clinical work; fully conversant, too, with the requirements of the licensing and examining bodies and of the Medical Council." He must also be a gentleman of scholarly attainments and of good presence. The University of Oxford fortunately possesses many graduates in medicine who combine all these requirements, and the choice would be one of great difficulty did not the question of stipend also come into account. The Chair of Medicine is very poorly endowed, scarcely £400 a year, if report speaks truly—a sum which is wholly inadequate to maintain the dignity of the position, and the selected candidate must either possess private means, or he must be in a position to augment his income by combining the professorship with some other university post. The University of Oxford, so far as it is represented by the science teachers, desire that the Regius Professor of Medicine should be actively engaged in teaching, and to this end they wish that the Readership in Pathology should be united, at any rate temporarily, with the Regius Professorship, and that the present Reader in Pathology, a capable and very highly distinguished teacher and original worker, should be appointed to the combined office. The scheme would be unimpeachable if an individual could be found equally eminent as a physician and as a pathologist, for the Regius Professor of Physic at Oxford or at Cambridge is much more than a mere teacher. He holds a very important position in the medical profession not only of this country, but throughout the world; his advice is often sought on questions of great importance; and whilst there is every reason for rescuing the Oxford professorship from any chance of becoming a sinecure, it should not be allowed to degenerate into a mere Chair of Pathology. The Oxford medical graduates, therefore, have acted very rightly in protesting formally against any attempt to "rush" the appointment. There is very little doubt that a man suitable in every respect can be found if a little time be given for reflection. Though, no doubt *erant aliqui, quibus si constaret quis futurus esset abbas, non ita devote orassent*, in the words of the Brother Jocelin: "There were some among us who, had it been known who was to be abbot, would not have prayed quite so devoutly."

